# Autologous monocyte-derived DC vaccination combined with cisplatin in stage III and IV melanoma patients: a prospective, randomized phase 2 trial

**DOI:** 10.1007/s00262-019-02466-x

**Published:** 2020-01-24

**Authors:** Steve Boudewijns, Martine Bloemendal, Nienke de Haas, Harm Westdorp, Kalijn F. Bol, Gerty Schreibelt, Erik H. J. G. Aarntzen, W. Joost Lesterhuis, Mark A. J. Gorris, Alexandra Croockewit, Lieke L. van der Woude, Michelle M. van Rossum, Marieke Welzen, Anna de Goede, Stanleyson V. Hato, Winette T. A. van der Graaf, Cornelis J. A. Punt, Rutger H. T. Koornstra, Winald R. Gerritsen, Carl G. Figdor, I. Jolanda M. de Vries

**Affiliations:** 1 Department of Medical Oncology, Radboud University Medical Center, Nijmegen, The Netherlands; 2 Department of Tumor Immunology, Radboud University Medical Center, Radboud Institute for Molecular Life Sciences, PO Box 9101, 6500 HB Nijmegen, The Netherlands; 3 Department of Pharmacy, Radboud University Medical center, Nijmegen, The Netherlands; 4 Department of Radiology and Nuclear Medicine, Radboud University Medical Center, Nijmegen, The Netherlands; 5 School of Biomedical Sciences, University of Western Australia, Crawley, Australia; 6 Department of Hematology, Radboud University Medical Center, Nijmegen, The Netherlands; 7 Department of Pathology, Radboud University Medical Center, Nijmegen, The Netherlands; 8 Department of Dermatology, Radboud University Medical Center, Nijmegen, The Netherlands; 9 Department of Medical Oncology, Academic University Medical Center, University of Amsterdam, Amsterdam, The Netherlands; 10 Oncological Center, Rijnstate Hospital, Arnhem, The Netherlands

**Keywords:** Dendritic cell, Vaccination, Cisplatin, Melanoma, Immunotherapy

## Abstract

**Background:**

Autologous dendritic cell (DC) vaccines can induce tumor-specific T cells, but their effect can be counteracted by immunosuppressive mechanisms. Cisplatin has shown immunomodulatory effects in vivo which may enhance efficacy of DC vaccination.

**Methods:**

This is a prospective, randomized, open-label phase 2 study (NCT02285413) including stage III and IV melanoma patients receiving 3 biweekly vaccinations of gp100 and tyrosinase mRNA-loaded monocyte-derived DCs with or without cisplatin. Primary objectives were to study immunogenicity and feasibility, and secondary objectives were to assess toxicity and survival.

**Results:**

Twenty-two stage III and 32 stage IV melanoma patients were analyzed. Antigen-specific CD8^+^ T cells were found in 44% versus 67% and functional T cell responses in 28% versus 19% of skin-test infiltrating lymphocytes in patients receiving DC vaccination with and without cisplatin, respectively. Four patients stopped cisplatin because of toxicity and continued DC monotherapy. No therapy-related grade 3 or 4 adverse events occurred due to DC monotherapy. During combination therapy, one therapy-related grade 3 adverse event, decompensated heart failure due to fluid overload, occurred. The clinical outcome parameters did not clearly suggest significant differences.

**Conclusions:**

Combination of DC vaccination and cisplatin in melanoma patients is feasible and safe, but does not seem to result in more tumor-specific T cell responses or improved clinical outcome, when compared to DC vaccination monotherapy.

**Electronic supplementary material:**

The online version of this article (10.1007/s00262-019-02466-x) contains supplementary material, which is available to authorized users.

## Introduction

Based on their capacity to activate and prime naïve T cells, dendritic cells (DCs) are the most efficient antigen-presenting cells of the immune system. This makes them ideal candidates to be exploited for vaccination therapies [3]. Studies with autologous DC vaccines have shown to induce tumor-specific immune responses in both lymph node involved (stage III) and metastatic (stage IV) melanoma patients [4]. Despite immunological responses, objective clinical responses are rare in stage IV melanoma patients [5–7]. In stage III patients, a higher percentage of immunological responses to DC vaccination are observed, which could be explained by a lower tumor burden and concomitant less tumor-induced immune suppression. Accordingly, we found favorable overall survival (OS) in stage III melanoma patients who received adjuvant DC vaccination compared to matched controls [8], which is currently investigated in our randomized phase 3 trial (NCT02993315).

Combination with therapies modulating an immunosuppressive tumor microenvironment (TME) may strengthen the effect of DC vaccination. Platinum-based chemotherapeutics are widely used for several types of cancer [9]. Cisplatin was also tested in metastatic melanoma patients, as monotherapy and in combination with other types of chemotherapy, interferon (IFN) or interleukin (IL)-2. However, without great clinical benefit and with more toxicity than dacarbazine, another chemotherapeutic, dacarbazine was the preferred systemic therapy at time of trial enrollment [10–13]. The rationale to combine DC vaccination with cisplatin is based on the ability of cisplatin to not only cross-link DNA and inhibit mitosis, but also to have immunomodulatory effects [14, 15]. In vitro platinum drugs cause inhibition of signal transducer and activator of transcription (STAT) signaling via decreasing phosphorylation of different STAT proteins [16]. STAT proteins each have a different effect on the anti-tumor response [17, 18]. For example, diminished STAT6 phosphorylation results in downregulation of the T cell inhibitory molecule programmed death ligand 2 (PD-L2) on both DCs and tumor cells, which enhances tumor cell recognition by T cells [15, 18]. More recently, preclinical studies showed that cisplatin may upregulate MHC class I expression on tumor cells and upregulate the lytic activity of cytotoxic effector cells [14]. Furthermore, it has been shown that cisplatin can improve recruitment of immune effector cells to the TME and enhances their proliferation and at the same time causes downregulation of immunosuppressive cells in the TME by reducing levels of myeloid-derived suppressor cells (MDSCs) and regulatory T cells (Tregs) [14, 19]. Besides this effect in the TME, a reduction in circulating Tregs was found in patients with non-small cell lung cancer after treatment with cisplatin and vinorelbine [20]. Finally, it was recently observed that cisplatin can induce immunogenic cell death [21].

In a preclinical tumor model, synergy was shown between vaccination with synthetic long peptides of human papillomavirus (HPV) type 16 and cisplatin. Combined treatment led to highly infiltrated tumors with HPV-specific tumor necrosis factor (TNF)-α and IFN-γ producing T cells and significantly decreased tumor cell proliferation compared to single treatment [22]. Combining DC vaccination with cisplatin may have a similar synergistic effect, as the immunomodulatory effects of cisplatin potentially improve the efficacy of the antigen-specific T cells induced by DC vaccination.

The aim of this study was to explore whether the combination of autologous DC vaccination and cisplatin in stage III and IV melanoma patients is feasible and safe and whether it leads to better immunological and clinical responses compared to DC monotherapy.

## Materials and methods

### Patient characteristics

Patients between 18 and 70 years of age with histologically confirmed stage III or IV melanoma, both with a cutaneous (American Joint Cancer on Committee (AJCC) 7th edition [23]) and uveal (AJCC 7th edition [24]) melanoma, were eligible. Additional key eligibility criteria included: WHO performance status of 0 or 1; melanoma expressing gp100 (compulsory) and tyrosinase (non-compulsory) as assessed by immunohistochemistry performed on previously obtained tissue; normal serum lactate dehydrogenase (LDH); life expectancy of at least 3 months; serum creatinine level < 150 μmol/L; within 2 months of radical lymph node dissection; and at least one measurable target lesion according to Response Evaluation Criteria in Solid Tumors (RECIST) version 1.1 in stage IV patients. Key exclusion criteria were: any prior chemotherapy, immunotherapy or radiotherapy within 4 weeks of the first vaccination; symptomatic brain metastases; rapidly progressive symptomatic disease; a history of any second malignancy in the previous 5 years; autoimmune disease; use of immunosuppressive drugs; and a known allergy to shell fish due to the use of Keyhole limpet hemocyanin (KLH) protein.

### Study design and treatment

In this open-label phase 2 study, patients were randomly assigned between two experimental arms in a 1:1 ratio to receive autologous DC vaccination with or without cisplatin, stratified for disease stage (III versus IV). Unresectable stage III was considered stage IV disease. Vaccines consisted of autologous cytokine-matured monocyte-derived DCs electroporated with mRNA encoding gp100 and tyrosinase. Patients received three biweekly vaccinations, followed by a delayed-type hypersensitivity (DTH) skin test (Supplementary Fig. 1). Patients received two additional cycles of vaccinations at 6-month intervals. Cisplatin (50 mg/m^2^, maximum of 100 mg/dose) was administered intravenously 1–2 h before DC injection. The dose was based on in vitro experiments, evaluating the effect on cytokine production of DCs [18]. As cisplatin is highly emetogenic, the standard antiemetic regime consisted of dexamethasone (10 mg intravenously on day 1 or 12 mg orally on day 1–4), aprepitant, ondansetron and metoclopramide. Since February 2014, this regime changed to 12 mg orally on day 1–4. Primary objectives of the study were to study immunological response and feasibility of the addition of cisplatin to DC vaccination. Therefore, patients were replaced when no DTH skin test was performed. Secondary objectives were to assess toxicity, recurrence-free survival (RFS), progression-free survival (PFS) and OS. Toxicity was assessed according to the National Cancer Institute Common Terminology Criteria for Adverse Events (CTCAE) version 3.0. Tumor evaluation was performed at baseline and every 3 months thereafter by physical examination in stage III patients and by a CT scan of chest and abdomen evaluated according to RECIST version 1.1 in stage IV patients. Treatment was stopped with disease recurrence (stage III), progression (stage IV), unacceptable toxicity or withdrawal of consent.

### Vaccine production

Monocytes were enriched from leukapheresis products as described before [25]. Monocytes were cultured in X-VIVO 15 medium (Lonza) supplemented with 2% human serum (HS; Sanquin), IL-4 (500 U/ml), GM-CSF (800 U/ml, both CellGenix) and KLH (10 μg/ml, Calbiochem). DCs were matured with a cocktail of 10 ng/ml TNF-α, 5 ng/ml IL-1β, 15 ng/ml IL-6 (all CellGenix) and prostaglandin E_2_ (10 μg/ml, Pharmacia & Upjohn) [26]. Cells used for the DTH skin test were cultured without KLH. DCs were electroporated with mRNA encoding gp100 or tyrosinase, as previously described [27]. Patients could only participate if the predefined phenotypic minimal release criteria used in clinical trials were met [28]. DCs were administered both intradermally (maximum of 10 × 10^6^ cells) and intravenously (maximum of 20 × 10^6^ cells).

### Flow cytometry

Phenotype of the ex vivo generated DCs was characterized by flow cytometry with the following monoclonal antibodies (mAbs): anti-HLA-ABC-PE, anti-HLA-DR-PE, anti-CD80-PE, anti-CD83-PE, anti-CD86-PE, anti-CD3-PE, anti-CD25-PE, anti-CD95-PE (all BD Biosciences), anti-CD14-PE (Sanquin Reagents), anti-HLA-DQ-PE, anti-CD20-PE (both BioLegend) and anti-CCR7-PE (Miltenyi Biotec). For intracellular staining, anti-gp100 (NKI/beteb; Netherlands Cancer Institute) and anti-tyrosinase (T311; Cell Marque Corp) were used. Flow cytometry was carried out using a FACSCalibur flow cytometer equipped with CellQuest software (BD Biosciences).

The presence of Tregs and monocytic (M)-MDSCs was analyzed in PBMCs isolated from heparinized blood collected prior to the apheresis and on the day of and prior to the first DTH skin test by Ficoll-Paque density centrifugation. The Treg antibody panel consisted of fixable viability dye 780, anti-FoxP3-Alexa488 (both eBioscience), anti-CD3-BV605 (BioLegend), anti-CD4-BV510 and anti-CD25-BV421 (both BD Biosciences). M-MDSCs were analyzed with anti-CD33-APC (BioLegend), anti-CD14-BV421, anti-HLA-DR-BV510 and anti-CD11b-BV605 (all BD Biosciences) antibodies. Flow cytometry was carried out using a FACSLyric equipped with FACSuite software (BD Biosciences). Tregs were identified as CD3^+^CD4^+^CD25^+^FoxP3^+^ cells. M-MDSCs were analyzed as HLA-DR^−^CD14^+^CD11b^+^CD33^+^ cells. Analyses were carried out using FlowJo software version 10.0.7 (Treestar Inc.).

### KLH-specific proliferation

PBMCs were stimulated with KLH (4 µg/2 × 10^5^ PBMC; Immucothel, Biosyn) in X-VIVO 15 with 2% HS. After 3 days, cells were incubated with ^3^H-thymidine for 8 h and incorporation was measured with a β-counter. Experiments were performed in sextuplicate, and ovalbumin was used as control. Response to KLH is given as proliferation index (proliferation with KLH/proliferation without KLH).

### Skin-test infiltrating lymphocyte culture analysis

One to two weeks after each vaccination cycle, skin tests were performed, as described previously [29]. In short, DCs electroporated with gp100 and/or tyrosinase (1 × 10^6^ DCs maximum in total) were thawed and injected intradermally at the back of patients at four different sites. After 2 days, punch biopsies (6 mm) were taken. Half of each biopsy was manually cut and cultured for 2–4 weeks in RPMI-1640 containing 7% HS and IL-2 (100 U/ml, Novartis).

Skin-test infiltrating lymphocyte (SKIL) cultures and PBMCs from HLA-A2.1 positive patients were stained with HLA-A2.1 tetrameric MHC complexes containing the epitopes gp100:154-162, gp100:280-288 or tyrosinase:369-377 (Sanquin) as described before [30]. Human immunodeficiency virus was used as negative control. Tetramer positivity (TM^+^) was defined as at least a twofold increase in the double positive population compared to control. In HLA-A2.1 positive patients, the production of IFN-γ was measured in supernatants after 16 h of co-culture with different target cells: T2 cells pulsed with gp100:154-162, gp100:280-288 or tyrosinase:369-377; BLM (HLA-A2.1-positive melanoma cell line without endogenous expression of gp100 and tyrosinase) transfected with gp100, tyrosinase or control antigen G250; and Mel624 (HLA-A2.1-positive, gp100-positive and tyrosinase-positive tumor cell line). Cytokine analysis was performed by cytometric bead array (CBA) (human Th1/Th2 FlowCytomix multiplex kit, eBioscience).

In HLA-A2.1-negative patients, cytokine production by SKILs was determined by using autologous Epstein–Barr virus (EBV)-transformed B cells as described by van Nuffel et al. [31]. Autologous EBV-B cells were generated from PBMCs and electroporated with mRNA encoding full-length gp100 or tyrosinase (Curevac GmbH). Carcinoembryonic antigen (CEA) was used as a negative control. MRNA-loaded EBV-B cells were co-cultured 1:1 with SKILs for 24 h. Afterward, expression of the early activation markers CD69, CD107a and CD137 on CD8^+^ T cells was analyzed. Phorbol myristate acetate-stimulated (5 μg/ml; Sigma-Aldrich) SKILs were used as positive control. After 24 h of co-culture, cytokine production was measured with CBA.

### Multiplex immunofluorescence staining

Tumor tissue resected prior to the start and after experimental therapy was collected if available. Sections of 4 μm from formalin-fixed paraffin-embedded (FFPE) tissue were deparaffinized, and sections from frozen tissue were dried and fixed in 4% formaldehyde.

Three-color multiplex immunohistochemistry (mIHC) using Opal 7-Color IHC Kit (NEL801001KT, Perkin Elmer) was performed for the detection of STAT3 and phosphorylated STAT3 (pSTAT3). After antigen retrieval, tissue sections were subjected to mAbs listed in Supplementary Table 1a.

A seven-color mIHC for the detection of lymphocyte populations was applied using the BOND RX IHC & ISH Research Platform (Leica Biosystems) with mAbs for CD45RO, CD8, CD20, CD3, Foxp3 and a melanoma mix as listed in Supplementary Table 1b. For analysis, CD3^+^CD8^−^ cells were considered CD4^+^ T cells. All epitope retrievals and antibody-TSA complex removals were performed using BOND Epitope Retrieval 2 (AR9640, Leica Biosystems). Blocking steps were performed with antibody diluent for 10 min, primary antibody incubations for 1 h, secondary antibody Opal polymer HRP Ms + Rb incubations for 30 min and Opal reagent incubations for 10 min, all at room temperature. Tissue was counterstained with 4′,6-diamidino-2-phenylindole (DAPI) and mounted with Fluoromount-G (0100-01; Southern Biotech). A similar seven-color mIHC was performed on cryopreserved tissue with anti-granzyme B instead of CD20 (Supplementary Table 1c).

The slides were scanned using the Automated Quantitative Pathology Imaging System Vectra 3.0.4. Regions of interest were selected using Phenochart version 1.0.9 for multispectral imaging at 20 × magnification. InForm version 2.2.1. was used for spectral unmixing of Opal fluorophores, DAPI and autofluorescence and downstream imaging analysis (all PerkinElmer Inc.).

For pSTAT3 analysis, the percentage of nuclei containing pSTAT3 was counted separately by two investigators. Divergent results were discussed to reach consensus. For lymphocyte analysis, a selection of 30–35 representative original multispectral images was used to train InForm to distinguish tumoral from stromal tissue and background based on DAPI and autofluorescence. Settings for adaptive cell segmentation were based on DAPI and membrane signals. All settings applied to the training images were saved in an algorithm to allow batch analysis. Segmented cell data were analyzed using FlowJo in which immune cells were phenotyped by manual gating and divided by the surface area of the tissue region (mm^2^).

### Statistical analysis

A sample size of 27 patients per arm was calculated using a two-sided log-rank test, to have 80% power with an alpha of 0.05 to detect an anticipated improvement from 30 to 70% in immunological response rate with the addition of cisplatin to DC vaccination. Survival was calculated from the date of apheresis to the first date of progression (PFS) in stage IV and recurrence (RFS) in stage III, using the Kaplan–Meier method. Difference between treatment groups was evaluated using a log-rank test. Recurrence and progression were censured in case of non-melanoma-related death. Follow-up duration was determined from date of apheresis to date of the last follow-up and censored for death. Immunological results after the first cycle were used to prevent a guarantee time bias. Paired *t* tests were performed to evaluate KLH responses before and after vaccination and independent-samples *t* tests to evaluate differences in KLH proliferation between groups. For TM^+^CD8^+^ T cells and functional T cells, differences between groups were evaluated using a Chi-square test or 2-sided Fisher’s exact test in case of expected count < 5. *p* values < 0.05 were considered significant. IBM SPSS Statistics version 25.0 (IBM Corp.) and Graphpad Prism 5.03 (GraphPad Software Inc.) were used for statistical analysis and data visualization.

## Results

### Patient and vaccine characteristics

Between February 2011 and July 2014, sixty patients were screened and included in the trial (Supplementary Fig. 2) of whom six were replaced: two stage IV patients because no acceptable DC product could be produced and four stage IV patients since they had progressive disease prior to the first immunological assessment. Therefore, 54 patients were included in the final analysis: 22 stage III and 32 stage IV melanoma patients. Patients were randomly assigned to receive either DC vaccination alone or combined with cisplatin. In all but one patient included, a DC product meeting the predefined minimal release criteria could be produced from the first apheresis (Supplementary Fig. 3a). In this particular patient, this was achieved after a repeated apheresis. Flow cytometry confirmed intracellular protein expression of both gp100 and tyrosinase in DCs (Supplementary Fig. 3b). In two patients, yield was insufficient for three vaccinations; therefore, apheresis was repeated during the first cycle.

Baseline characteristics of immunologically evaluable patients are summarized in Table 1. Overall, in the stage III group, five patients (23%) had stage IIIA, 5 (23%) had stage IIIB, and 11 (50%) had stage IIIC disease. Most patients (73%) with IIIC melanoma were randomized to receive monotherapy. Eleven patients (50%) completed all three cycles of three vaccinations, seven patients receiving combination therapy and four patients with DC monotherapy.

**Table 1:** Baseline characteristics

	Stage III melanoma patients		Stage IV melanoma patients	
	DC vaccination (*n* = 11)	DC vaccination + cisplatin (*n* = 11)	DC vaccination (*n* = 16)	DC vaccination + cisplatin (*n* = 16)
Sex, *n* (%)				
Male	9 (82)	9 (82)	8 (50)	10 (63)
Female	2 (18)	2 (18)	8 (50)	6 (38)
Age (years)—median (range)	53 (25–69)	48 (25–67)	61 (34–69)	54 (30–69)
HLA-A2.1, *n* (%)				
Positive	7 (64)	9 (82)	5 (31)	9 (56)
Negative	4 (36)	2 (18)	11 (69)	7 (44)
Site of primary melanoma, *n* (%)				
Skin	10 (91)	10 (91)	12 (75)	12 (75)
Eye	0 (0)	0 (0)	3 (19)	1 (6)
Unknown primary	1 (9)	0 (0)	1 (6)	3 (19)
Primary not assessed	0 (0)	1 (9)	0 (0)	0 (0)
AJCC stage (7th edition)^a^, *n* (%)				
IIIA	2 (18)	3 (27)	n.a	n.a
IIIB	1 (9)	4 (36)		
IIIC	8 (73)	3 (27)		
IIIX	0 (0)	1 (9)		
Adjuvant radiotherapy, *n* (%)				
No	7 (64)	8 (73)	n.a	n.a
Yes	4 (36)	3 (27)		
M stage at inclusion, *n* (%)				
M0	n.a	n.a	1 (6)	1 (6)
M1a			3 (19)	4 (25)
M1b			5 (31)	4 (25)
M1c			7 (44)	7 (44)
Prior treatment for stage IV disease, *n* (%)				
No	n.a	n.a	7 (44)	12 (75)
Surgery			8 (50)	3 (19)
Radiotherapy			1 (6)	0 (0)
Targeted therapy			1 (6)	0 (0)
Chemotherapy			1 (6)	0 (0)
Regional perfusion			0 (0)	2 (13)

^a^The appropriate American Joint Committee on Cancer (AJCC) TNM system was used for both cutaneous (7th edition [23]) and uveal (7th edition [24]) melanomas

The stage IV group included 26 patients (81%) with metastatic cutaneous melanoma, 2 (6%) with irresectable stage III disease and 4 (13%) with metastatic uveal melanoma. Only five patients (16%) completed two cycles, and thereof, 2 (6%) completed the total of three cycles of vaccinations. One patient in the combination group with stable disease at the first evaluation scan at 3 months was referred for palliative surgical resection of a stable ileal metastasis to lower the risk of a gastrointestinal bleeding. At clinical data cutoff April 23, 2019, median follow-up was 62.3 months in stage III and 64.9 months in stage IV patients.

### Adverse events

All evaluable patients (*n* = 54) were included in the safety analysis (Table 2). The remaining four patients who did not complete at least one cycle showed no striking features in their toxicity profile. In the combination group, frequent adverse events included nausea and fatigue. One treatment-related grade 3 adverse event occurred, consisting of decompensated heart failure due to fluid overload. Cisplatin was stopped because of adverse events in four patients (15%) based on decompensated heart failure, a deep venous thrombosis, persistent grade 2 tinnitus and grade 2 nausea and fatigue. DC vaccination was continued as monotherapy in all four patients. The dose of cisplatin was reduced in one patient after vaccination five because of grade 2 nausea. Treatment-related adverse events leading to dose interruptions occurred in two patients treated with cisplatin; due to grade 2 tinnitus or grade 2 thrombocytopenia. In the group treated with DC monotherapy, the most frequent adverse events were flu-like symptoms usually lasting less than 48 h and injection site reactions. No grade 3–4 events were observed.

**Table 2:** Treatment-related adverse events

	Number of events (%)					
	DC vaccination (*n* = 27)		DC vaccination + cisplatin (*n* = 27)			*p* value
	Grade 1	Grade 2	Grade 1	Grade 2	Grade 3	
Any event	22 (81)	2 (7)	18 (67)	7 (26)	1 (4)	0.159
Injection site reaction	20 (74)	1 (4)	10 (37)	0	0	0.008
Flu-like symptoms	19 (70)	3 (11)	17 (63)	0	0	0.092
Nausea	4 (15)	0	15 (56)	3 (11)	0	< 0.001
Vomiting	4 (15)	0	1 (4)	3 (11)	0	0.091
Creatinine increased	2 (7)	0	3 (11)	0	0	0.639
Constipation	0	0	7 (26)	0	0	0.005
Fatigue	0	0	4 (15)	3 (11)	0	0.018
Tinnitus	0	0	3 (11)	2 (7)	0	0.064

Adverse events that occurred in at least 10% of patients and were classified as possibly, probably or definitely related to the treatment by the investigator are depicted

### Induction of de novo immune responses

**Fig. 1:**
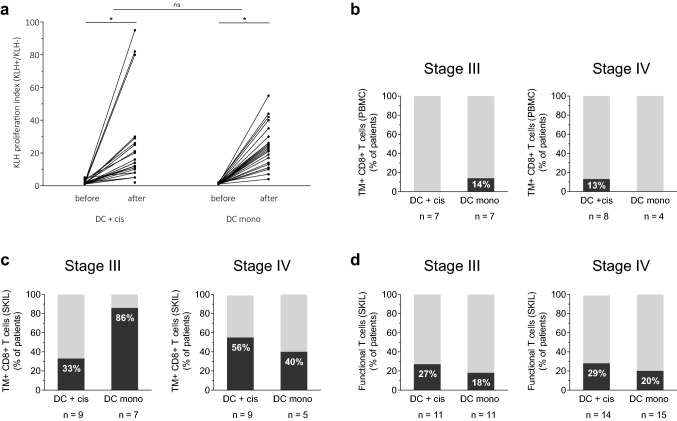
Immunological responses. **a** KLH-specific T cell proliferation was measured before the start of therapy and after each vaccination of the first cycle in PBMCs of melanoma patients. The proliferative response to KLH is depicted as the highest proliferation index (proliferation with KLH/proliferation without KLH) observed during the first cycle. **b** PBMCs were tested for TM^+^ CD8^+^ T cells recognizing gp100 or tyrosinase in HLA-A2.1 positive patients. **c** SKILs were tested for TM^+^ CD8^+^ T cells recognizing gp100 or tyrosinase in HLA-A2.1 positive patients and **d** a functional T cell response in all patients. *p < 0.001, *DC* dendritic cell, *KLH* Keyhole limpet hemocyanin, *ns* not significant, *PBMC* peripheral blood mononuclear cell, *SKIL* skin-test infiltrating lymphocytes, *TM* tetramer

DCs were loaded with the control antigen KLH, to test the capability to induce de novo immune responses. PBMCs after consecutive vaccinations of the first cycle showed an increase in KLH-specific T cell proliferation compared to baseline in all patients without significant difference in mean increase between both treatment groups (*p* = 0.453; Fig. 1a). In addition, no significant difference in mean increase was seen between stage III and IV patients (data not shown).

### Induction of tumor antigen-specific T cells

The presence of gp100- and tyrosinase-specific CD8^+^ T cells was tested with HLA-A2.1 tetrameric MHC–peptide complexes in both PBMCs and T cells cultured from biopsies of DTH injection sites (SKILs) of HLA -A2.1 positive patients. Eighteen HLA-A2.1-positive patients received combination therapy and 12 HLA-A2.1-positive patients DC monotherapy. TM^+^CD8^+^ T cells were found in PBMCs of two patients, one in each treatment group (Fig. 1b).

TM^+^CD8^+^ T cells were found more frequently in SKILs, in 16 out of 30 patients (53%) (Fig. 1c). There was no difference between treatment groups: 44% in the combination and 67% in the monotherapy group. In stage III patients, 33% treated with combination therapy compared to 86% treated with DC monotherapy showed TM^+^CD8^+^ SKILs. In stage IV patients, 56% of patients in the combination group compared to 40% in the monotherapy group showed TM^+^CD8^+^ SKILs. Regardless of the treatment arm, TM^+^CD8^+^ SKILs were found in 56% of stage III and 50% of stage IV patients.

SKILs were analyzed for the occurrence of a functional T cell response, by measuring production of IFNγ in response to cells loaded with gp100 or tyrosinase. This was found in 12 out of 51 patients (24%) tested (Fig. 1d), without difference between patients treated with combination therapy (28%) or DC monotherapy (19%).

### STAT expression and T cell infiltrate in tumor tissue

Tumor samples prior to and after DC vaccination were compared. Samples were not derived from the same tumor site, but we only used tissue of the same organ of origin, to obtain the best possible comparability. There were no clear differences in expression of nuclear pSTAT3 changes prior to and after DC vaccination between patients treated with monotherapy compared to combination therapy (Supplementary Fig. 4a, b). Therefore, we investigated other possible effects of cisplatin, such as decreasing numbers of Tregs in the TME. With mIHC, we investigated the presence of CD8^+^ T cells, CD4^+^ T cells and FoxP3^+^ cells. In these samples, no differences were observed between patients treated with or without cisplatin (Supplementary Fig. 4c–e).

**Fig. 2:**
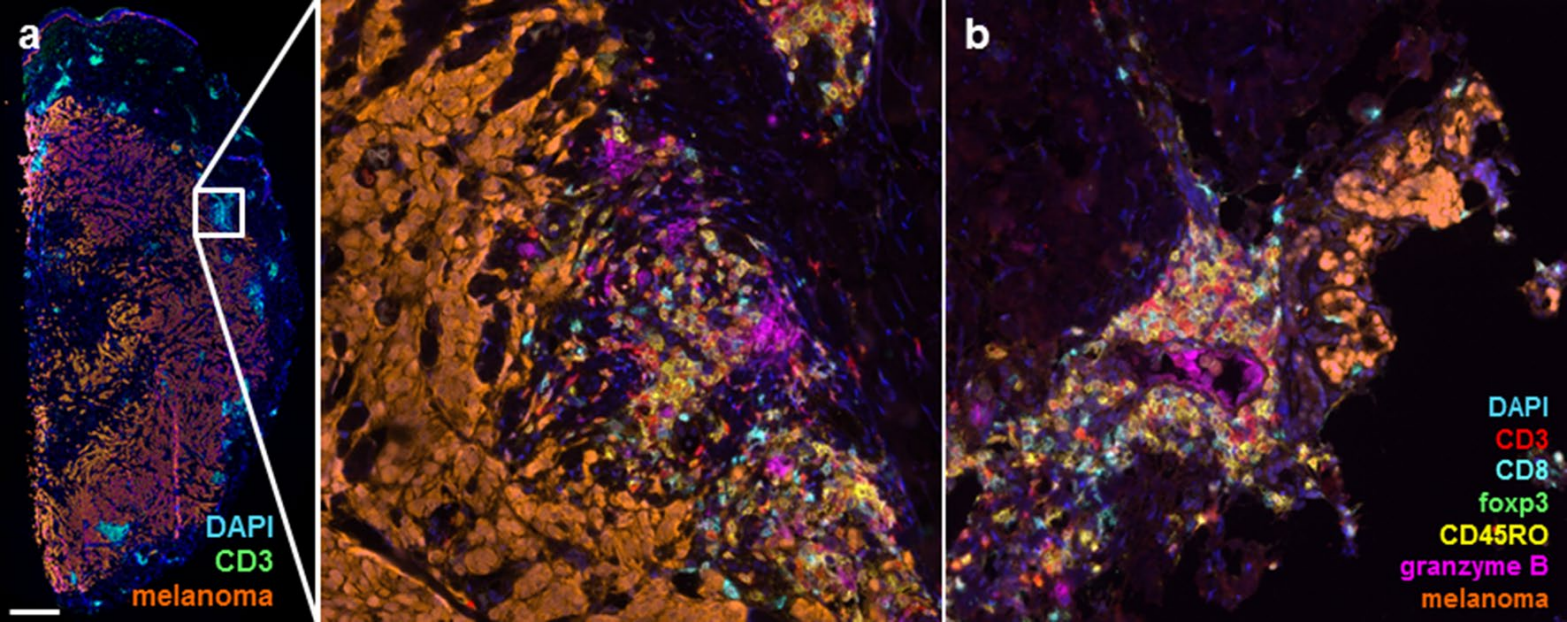
T cell infiltration in melanoma metastases. Multispectral images of cutaneous metastases were taken at the start and during treatment in a patient with a partial response to dendritic cell vaccination in combination with cisplatin. **a** Metastasis at the start of treatment shows extensive melanoma cells with groups of CD3^+^ cells. When zoomed in (middle image), it reveals that CD8^+^ T cells and granzyme B were present at the start of treatment. **b** Image of a clinically responding cutaneous metastasis after the second cycle of vaccinations combined with cisplatin, showing only few melanoma cells while an extensive T cell infiltrate was found, including CD8^+^ T cells and CD45RO^+^ cells

Of one patient with a partial response to combined treatment of DC vaccination with cisplatin, sequential in-transit metastases were investigated (Fig. 2). This patient did not show a functional T cell response in SKILs, but showed clear development of tumor necrosis and expanding T cell infiltration in a clinically responding metastasis during treatment.

Due to the retrospective nature of the tissue collection, only patients with recurrent or progressive disease were included in the immunohistochemistry analysis. To address this caveat, we analyzed PBMCs for the presence of M-MDSCs and Tregs at baseline and after three vaccinations, as this was also available for patients without recurrent disease. No differences in the presence of M-MDSCs or Tregs between stage III melanoma patients treated with or without cisplatin were found (Supplementary Fig. 5).

### Clinical response

**Fig. 3:**
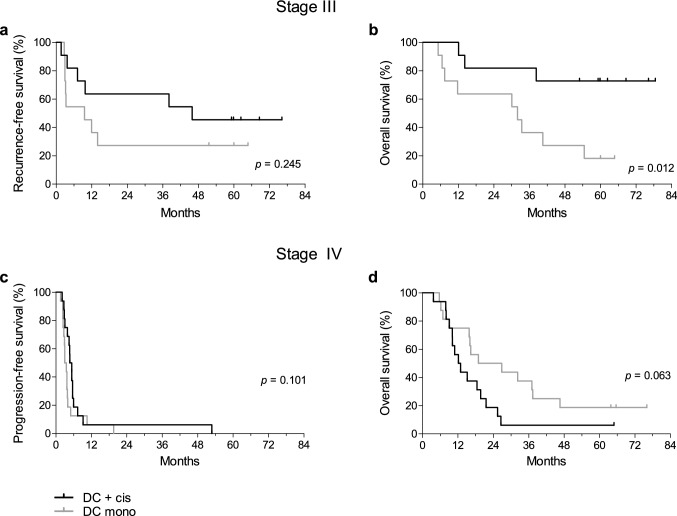
Clinical responses in stage III and IV melanoma patients. Kaplan–Meier curves for **a** recurrence-free survival and **b** overall survival in stage III melanoma patients. For stage IV melanoma patients, Kaplan–Meier curves for **c** progression-free survival and **d** overall survival are shown

Median RFS of stage III patients in the combination treatment group was 45.9 months versus 9.6 months in the monotherapy group (*p* = 0.245; Fig. 3a). The median OS of stage III patients treated with cisplatin was not reached, as compared to 32.0 months without cisplatin (*p* = 0.012; Fig. 3b). One patient in the monotherapy group died because of a non-melanoma-related cause without evidence of recurrent disease.

Median PFS of stage IV patients in the combination group was 4.7 months, as compared to 3.0 months in the monotherapy group (*p* = 0.101; Fig. 3c). When excluding metastatic uveal melanoma patients (one receiving cisplatin and three monotherapy), median PFS was 5.4 months in the combination group versus 3.5 months in the monotherapy group (*p* = 0.121). Stage IV patients treated with monotherapy had a trend toward a longer OS with a median of 19.0 versus 12.2 months in the combination group (*p* = 0.063; Fig. 3d). However, when excluding patients with uveal melanoma, this trend diminished. Subsequent treatment may have caused differences in OS, as more patients with progressive disease in the monotherapy than combination group received treatment with ipilimumab (69% versus 31%) or anti-PD1 antibodies (38% versus 0%).

## Discussion

In this randomized phase 2 trial, we showed that combining autologous DC vaccination with cisplatin is feasible and safe. Viable DCs, meeting the minimal release criteria [28], could be produced in 97% of patients. The toxicity profile of the combination treatment showed no unexpected safety concerns. Adverse events leading to discontinuation, interruption or dose reduction of cisplatin took place in a minority of patients. As expected, grade 1–2 injection site reactions and flu-like symptoms were common adverse events in both treatment groups, but occurred less in the combination group. These side effects might have been suppressed by dexamethasone, which was given as an antiemetic drug with cisplatin.

In this limited number of patients, the addition of cisplatin did not result in significantly improved immunological response to DC vaccination. In both treatment groups, DC vaccines induced de novo immune responses, despite the use of dexamethasone in the combination group. TM^+^CD8^+^ SKILs were induced in about half of the patients without difference between the treatment groups, although in stage III melanoma patients, combination therapy might have induced less TM^+^CD8^+^ T cells than monotherapy.

The lack of improved immunological response with addition of cisplatin might be explained by several reasons. It is possible that the immunomodulatory effects of cisplatin in the dosage and regimen used are not strong enough to enhance the number of anti-tumor T cell responses in vivo significantly. Although we saw a clear increase in T cell infiltration in the metastasis of one responding patient to the combination treatment, we found little other evidence on the in vivo immunostimulatory effect of cisplatin. The timing of tumor sampling relative to the dosing of cisplatin might also be suboptimal, as the interval between the last dose of cisplatin and retrieval of tumor tissue was at least a few weeks. In previous studies, the in vitro effects on STAT expression were seen immediately after exposure to platinum drugs [16, 18]. In addition, we investigated possible effects of cisplatin on the composition of the tumor immune infiltrate, showing no clear differences between treatment groups. Possibly explained by the retrospective nature of the collection of tumor material which retrieved only tissue of patients with  progressive or recurrent disease. As pSTAT3 upregulation is associated with tumor proliferation, this is a probable explanation of the increased pSTAT3 expression after vaccination in all but two patients. Prospectively collected tumor biopsies are of interest for better comparability in both responders and non-responders.

Dexamethasone could have had a negative effect on response induction in the combination group. Glucocorticosteroids can decrease the number of circulating T cells and increase the proliferation of Tregs [32, 33]*.* Therefore, dexamethasone might have hampered both the sensitivity of our immunomonitoring tests and the enhancing effects of cisplatin on the anti-tumor immune response. Also, the timing of the DC vaccination in relation to cisplatin might have caused the lack of synergy. In contrast to our study, Welters et al. found that carboplatin–paclitaxel every 3 weeks resulted in vigorous vaccine-induced T cell responses in advanced cervical cancer patients when a single dose of HPV16 synthetic long peptide vaccine was given 2 weeks after the second cycle of chemotherapy. This was despite use of 20 mg dexamethasone intravenously as premedication. Their study showed that the decrease in circulating myeloid cells was most pronounced starting 2 weeks after the second cycle of chemotherapy, resulting in an optimal immunological window for vaccination [34]. Although these studies differ in tumor type, chemotherapy and type of vaccine, the most important difference could be the interval between chemotherapy and vaccination. In our study, this probably was too short to result in enhanced responses.

In this limited number of patients, we could not find a clear positive effect of the combination treatment on survival. A significant better OS is observed in stage III patients treated with combination therapy compared to DC monotherapy. However, groups are small and baseline characteristics too heterogenous to draw firm conclusions. For example, more stage IIIC patients were randomized in the DC monotherapy group. In addition, the difference in OS might be caused by ongoing responses to salvage therapy with immune checkpoint inhibitors (ICI) in two patients in the combination group while in the monotherapy group none responded to ICI. In addition, the clinical benefit was not supported by an increase in a clear increase in T cell responses. Finally, in stage IV, no significant difference in survival was found. Taken together, our data do not clearly suggest that addition of cisplatin to DC vaccination is of benefit to melanoma patients in the treatment schedule used.

On the other hand, cisplatin in combination with dexamethasone did not seem to harm immunological responses. More research is needed to optimize dosage and timing of cisplatin to assess its potential to enhance DC vaccination in vivo. Currently, ICI are available, and today, a study combining them with DC vaccination would, in melanoma patients, be preferred over combination with chemotherapy [35]. Combination might intensify proliferation and effector functions of tumor-specific T cells induced by DC vaccination by blocking inhibitory immune checkpoints with anti-CTLA-4 or anti-PD-1 mAbs [36, 37]. A recent phase 2 trial with DCs combined with anti-CTLA-4 mAbs showed tolerability and an encouraging objective response rate (38%) in pre-treated advanced melanoma patients [38]. Further clinical trials, mainly with the less toxic and more effective anti-PD-1 mAbs, are under investigation, and results are awaited [39].

In conclusion, combination of autologous monocyte-derived DC vaccination and cisplatin in stage III and IV melanoma patients is feasible and safe, but enhancement of the tumor-specific T cell responses or clinical benefit when compared to DC monotherapy could not be confirmed in this limited number of patients. However, together with the currently available ICI, future research in melanoma patients should focus on the more promising combination of DC vaccination with ICI.

### Electronic supplementary material

Below is the link to the electronic supplementary material.

Supplementary file1 (PDF 598 kb)

## Abbreviations

AJCC: American Joint Cancer on Committee
CBA: Cytometric bead array
CTCAE: Common terminology criteria for adverse events
DTH: Delayed-type hypersensitivity
EBV: Epstein–Barr virus
FFPE: Formalin-fixed paraffin embedded
HS: Human serum
ICI: Immune checkpoint inhibitors
KLH: Keyhole limpet hemocyanin
M-MDSC(s): Monocytic myeloid-derived suppressor cell(s)
mIHC: Multiplex immunohistochemistry
PD-L2: Programmed death ligand 2
pSTAT: Phosphorylated signal transducer and activator of transcription
RFS: Recurrence-free survival
SKIL(s): Skin-test infiltrating lymphocyte(s)
TM: Tetramer
