# Supplementary material for: Autologous monocyte-derived DC vaccination combined with cisplatin in stage III and IV melanoma patients: a prospective, randomized phase 2 trial
Source: Cancer Immunol Immunother. 2020 Jan 24;69(3):477–88. doi: 10.1007/s00262-019-02466-x (PMC7044256; doi:10.1007/s00262-019-02466-x)
Supplement: Supplementary file 1 — Supplementary file1 (PDF 598 kb) [file 262_2019_2466_MOESM1_ESM.pdf]

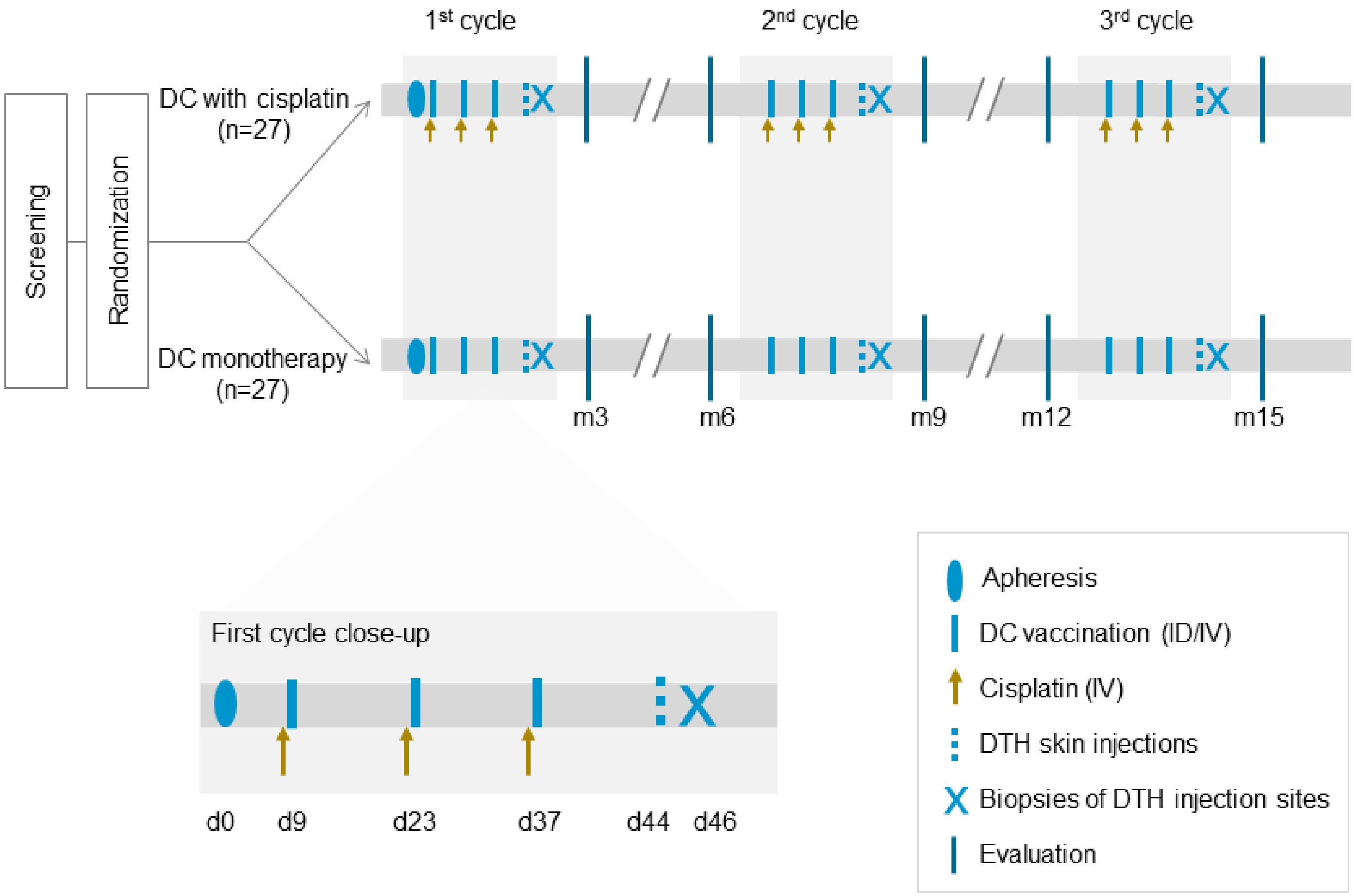

Supplementary Figure 1 Study schedule during the treatment phase

Patients were randomized between DC vaccination with cisplatin and DC vaccination monotherapy. Standard evaluation in stage IV patients consisted of CT of chest and abdomen and in stage III patients of medical history and physical examination. *D* day, *DC* dendritic cell, *DTH* delayed type hypersensitivity, *ID* intradermally, *IV* intravenously, *m* month

Supplementary Table 1a

Antibody	Fluorescence	Clone (catalogus number)	Dilution	Manufacturer
Anti-STAT3	Opal 650	79D7 (4904P)	1:100	Cell Signaling
Anti-pSTAT3	Opal 520	D3A7 (9145L)	1:100	Cell Signaling

Supplementary Table 1b

Antibody	Fluorescence	Clone (catalogus number)	Dilution	Manufacturer
Anti-CD45RO	Opal 620	UCHL-1 (MS-112)	1:1000	Thermo Fisher
Anti-CD8	Opal 690	C8/144B (M7103)	1:200	Dako
Anti-CD20	Opal 570	L26 (MS-340-S)	1:600	Thermo Fisher
Anti-CD3	Opal 520	Sp7 (RM-9107)	1:200	Thermo Fisher
Anti-Foxp3	Opal 540	236A/E7 (14-4777)	1:100	eBioscience Affymetrix
Melanoma mix				
HMB-45		HMB45 (M063401)	1:600	Dako
Mart-1	All Opal 650	A103 (MS-799)	1:300	Thermo Immunologic
Tyrosinase		T311 (MONX10591)	1:200	Monosan
SOX-10		EP268 (383R)	1:500	Cell Marque

Supplementary Table 1c

Antibody	Fluorescence	Clone (catalogus number)	Dilution	Manufacturer
Anti-CD45RO	Opal 620	UCHL-1 (MS-112)	1:1000	Thermo Fisher
Anti-Granzyme B	Opal 690	EPR8260 (ab134933)	1:300	Abcam
Anti-CD8	Opal 570	C8/144B (M7103)	1:1600	Dako
Anti-CD3	Opal 520	Sp7 (RM-9107)	1:200	Thermo Fisher
Anti-Foxp3	Opal 540	236A/E7 (14-4777)	1:100	eBioscience Affymetrix
Melanoma mix				
HMB-45		HMB45 (M063401)	1:600	Dako
Mart-1	All Opal 650	A103 (MS-799)	1:300	Thermo Immunologic
Tyrosinase		T311 (MONX10591)	1:200	Monosan
SOX-10		EP268 (383R)	1:500	Cell Marque

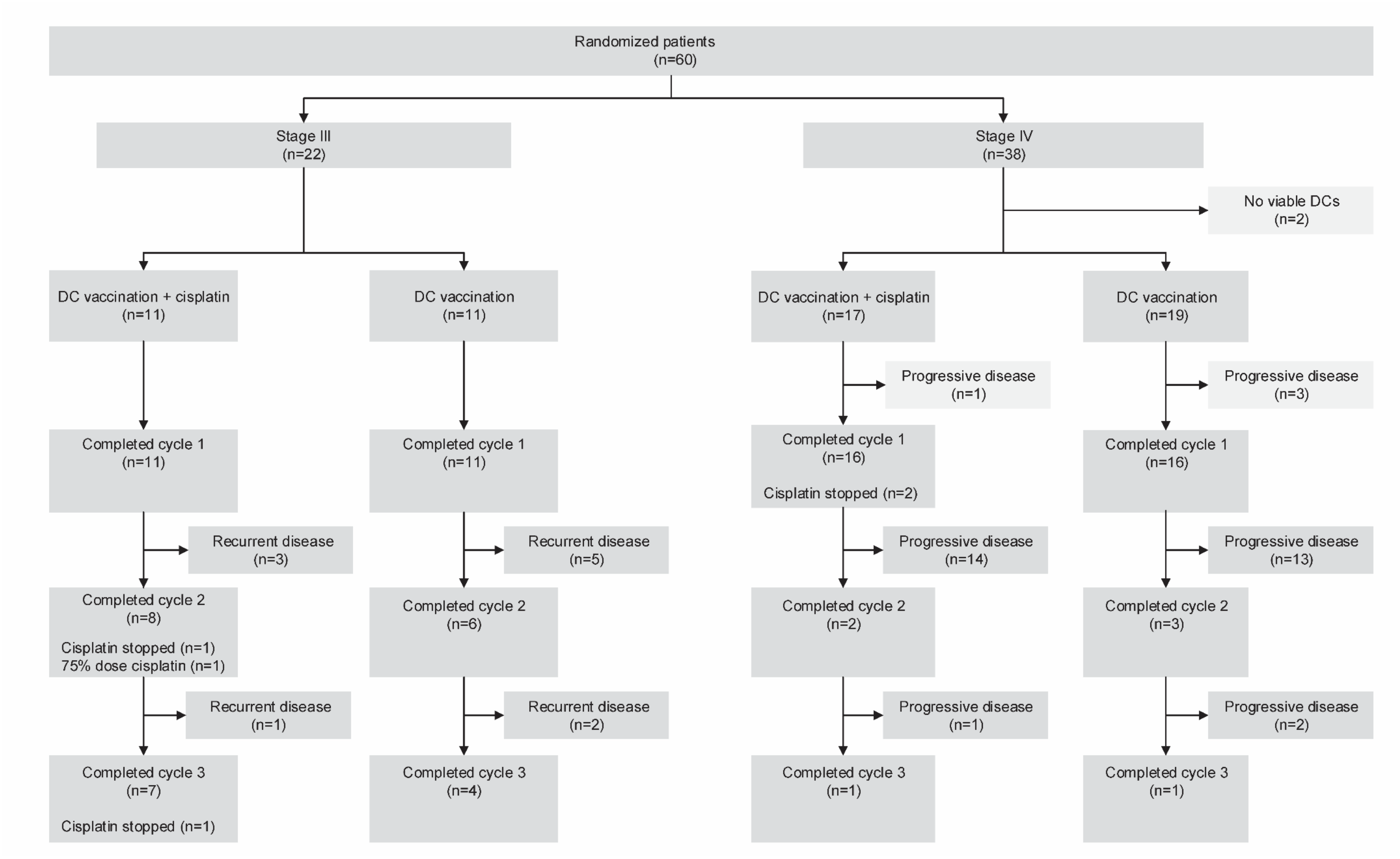

Supplementary Figure 2 Flowchart of all randomized stage III and stage IV melanoma patients

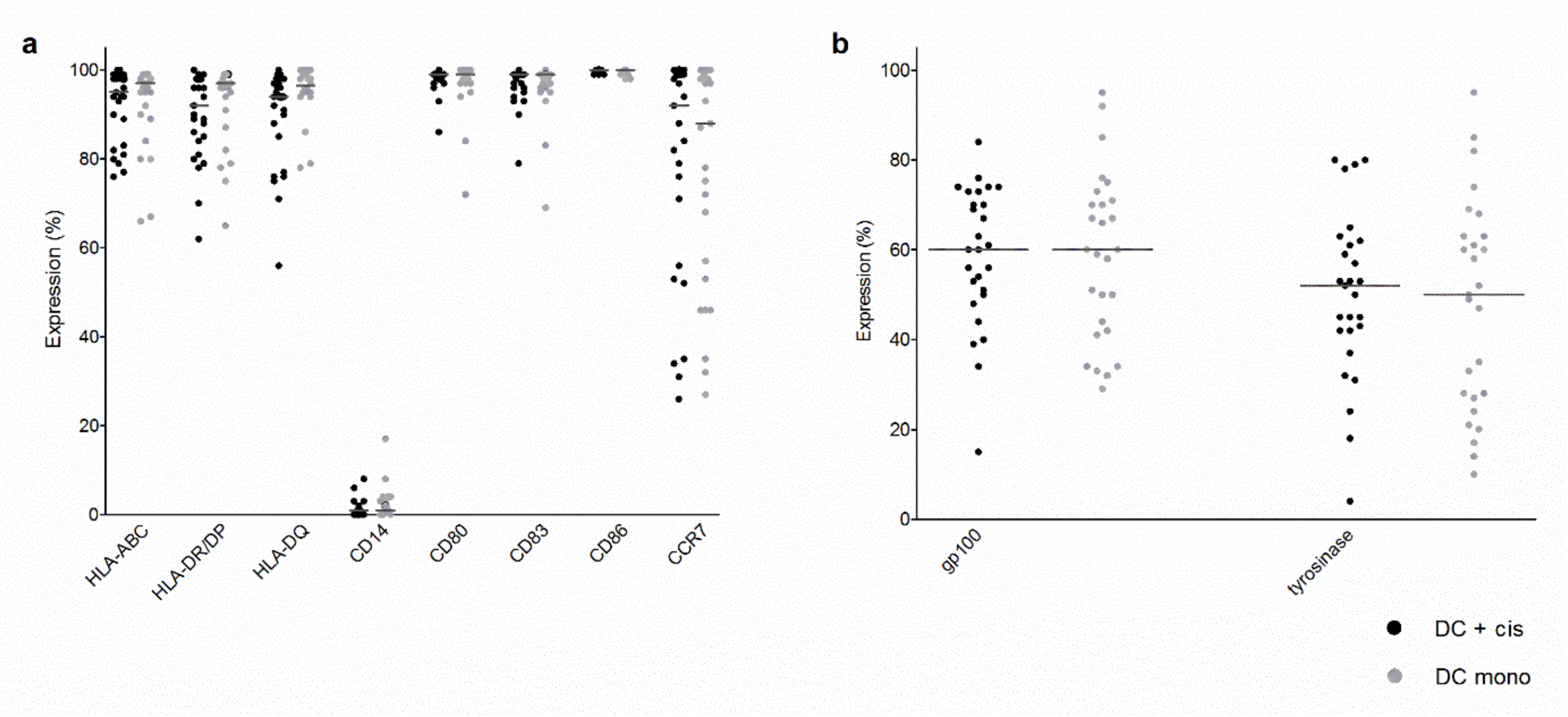

Supplementary Figure 3 Vaccine characteristics
Expression patterns of the *ex vivo* generated dendritic cells used in the first cycle. Shown are the percentages of dendritic cells expressing the (**a**) different phenotype markers and (**b**) tumor-associated antigens. The lines represent the median.

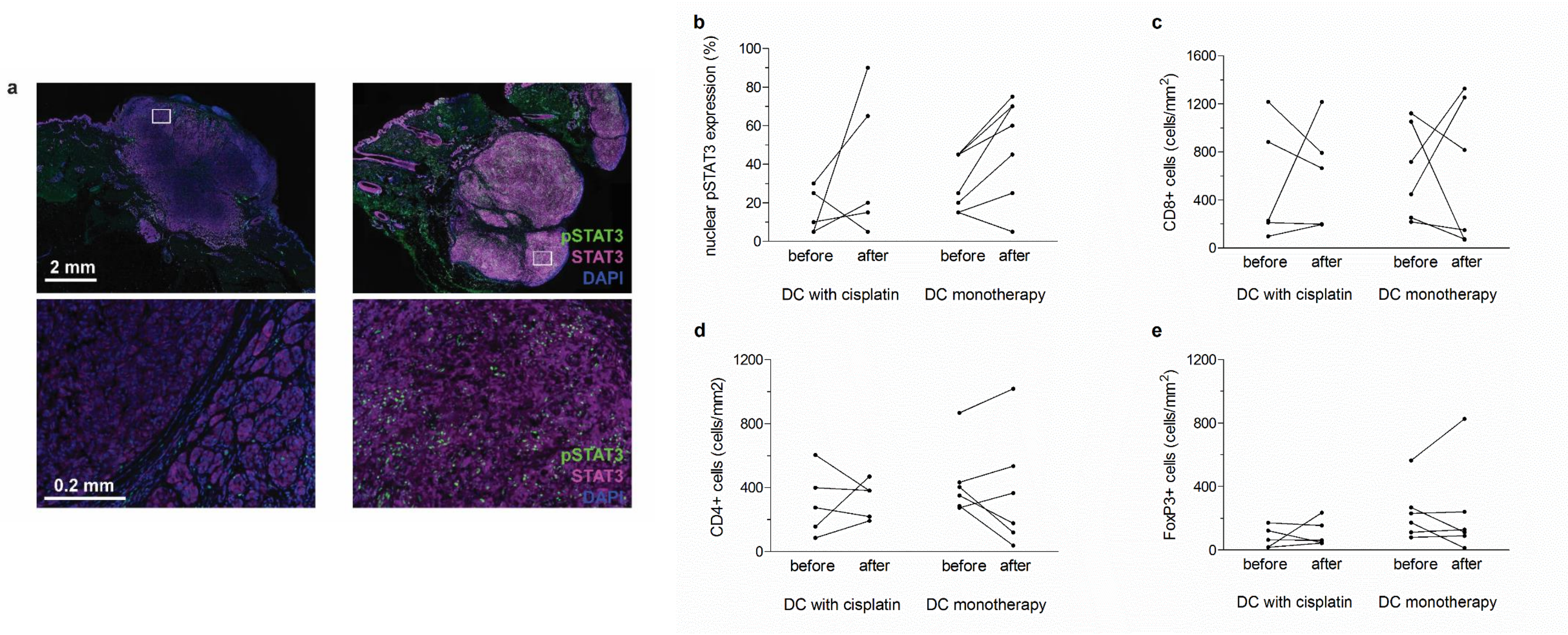

Supplementary Figure 4 pSTAT3 expression and infiltrate in melanoma tissue
Analyzed prior to and after experimental therapy. (**a**) Representative image of pSTAT3 staining by multiplex immunohistochemistry of low (**left**) and high (**right**). (**b**) Expression of nuclear pSTAT3. Analysis of lymphocyte infiltrate with absolute numbers of (**c**) CD8$^+$, (**d**) CD4$^+$ cells and (**e**) FoxP3$^+$ cells per mm^2.

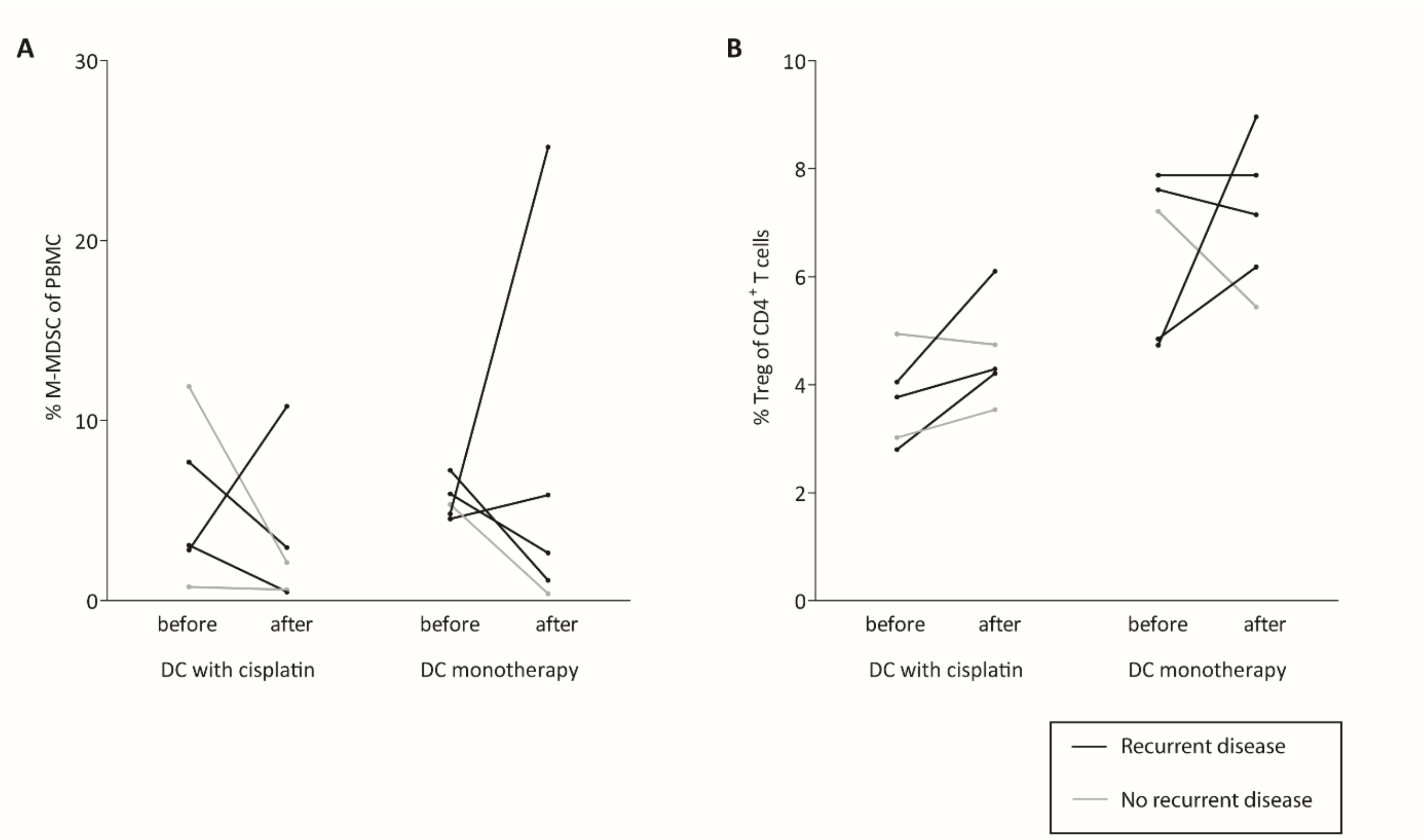

Supplementary Figure 5 Monocytic myeloid-derived suppressor cells (M-MDSCs) and regulatory T cells (Tregs) in peripheral blood. Peripheral blood mononuclear cells (PBMCs) were collected prior to the apheresis (start of study) and one week after the third vaccination. (**a**) percentage of M-MDSCs (HLA-DR$^-$CD14$^+$CD11b$^+$CD33$^+$) of live PBMCs and (**b**) the percentage of Tregs of CD4$^+$ T cells was determined for stage III patients treated with DC vaccination with or without concomitant cisplatin.
